# Multi-Omics Profiling of mTBI-Induced Gut–Brain Axis Disruption: A Preliminary Study for Biomarker Screening and Mechanistic Exploration

**DOI:** 10.3390/biomedicines14020311

**Published:** 2026-01-30

**Authors:** Xianqi Zhang, Tingting Wang, Yishu Liu, Shilin Miao, Pei Liu, Yadong Guo, Jifeng Cai, Changquan Zhang

**Affiliations:** Department of Forensic Science, School of Basic Medical Sciences, Central South University, Changsha 410013, China

**Keywords:** mild traumatic brain injury, gut microbiome, metabolome, multi-omics analysis, forensic identification

## Abstract

**Background/Objectives:** Mild Traumatic Brain Injury (mTBI) is a prevalent form of cranial trauma that can elicit a range of acute and chronic neuropsychiatric symptoms, and may increase the risk of neurodegenerative diseases. Its accurate identification remains a significant challenge in the field of forensic medicine. This study aimed to identify differential gut microbiota as potential biomarkers following mTBI and to preliminarily explore the association between alterations in gut microbiota and brain metabolites. **Methods:** An animal model was used to induce mTBI in male Sprague-Dawley (SD) rats. Dynamic changes in the gut microbiota and brain metabolites were analyzed via 16S rRNA sequencing and untargeted metabolomics. **Results:** Key discriminative taxa included *Staphylococcus*, *Streptococcus*, and *Aeromonadaceae*. Concurrently, brain metabolites, such as C24:1 Sphingomyelin and Thioetheramide PC, exhibited significant alterations. Multi-omics integration revealed that these changes were strongly correlated; in addition, a pathway analysis implicated disruptions in short-chain fatty acid and glycerophospholipid metabolism, which were linked to the regulation of inflammatory factors. **Conclusions:** This study demonstrates that mTBI induces distinct, time-dependent alterations in both the gut microbiota and brain metabolome, thereby providing a novel direction for research into the forensic diagnosis and mechanistic investigation of mTBI. Future studies are warranted to validate these potential biomarkers in human cohorts and to further elucidate the causal mechanisms underlying gut–brain axis interactions.

## 1. Introduction

Mild Traumatic Brain Injury, also referred to as concussion, is a common form of mechanical brain injury. Although considered minor in severity, it constitutes a significant risk factor for various chronic complications [1]. In the acute phase after mTBI, patients may experience symptoms such as headache, dizziness, and transient loss of consciousness. Over time, some individuals may develop neuropsychiatric symptoms including anxiety, depression, and attentional deficits [2], alongside an increased risk of neurodegenerative disorders such as Alzheimer’s disease (AD) and Parkinson’s disease (PD) [3], particularly after repetitive injuries [4]. Forensically, mTBI is commonly encountered in cases involving abuse, brawls, and traffic accidents. Evidence suggests a correlation between Traumatic Brain Injury (TBI) and elevated criminal behavior rates [5]. Legally speaking, the accurate determination of injury timing and severity assessment is critical for establishing causality and determining the nature of the case, which in turn is fundamental to ensuring equitable legal outcomes. However, the generally mild and nonspecific clinical manifestations of mTBI make its identification challenging. Therefore, there is a pressing need for research that can provide objective clues. Positioned as a preliminary step, this study aims to fill this gap by screening for biomarker candidates and exploring the initial mechanisms involved in mTBI.

Microorganisms constitute a vital component of human life. They colonize the skin, oral cavity, reproductive tract, and gastrointestinal tract of the host, playing roles in health maintenance, development, disease, and aging [6,7,8]. Current research on central nervous system (CNS) disorders, such as AD and PD, has revealed an association with dysbiosis of the gut microbiota, manifesting as reduced fecal microbial diversity, decreased abundance of beneficial bacteria *(Eubacterium rectale*, *Bifidobactrium)*, and increased abundance of potentially pathogenic microorganisms (*Bacteroides*, *Ruminococcus*, *Escherichia*/*Shigella)* [9]. Studies involving fecal microbiota transplantation (FMT) from healthy donors to injured recipients have demonstrated that the gut microbiota can alleviate and repair certain symptoms of CNS diseases [9,10,11], with this gut–brain interaction mediated through bidirectional communication along the gut–brain axis [7]. Microbial metabolites interact with the host environment, modulate immune responses via mucosal interfaces, enter the bloodstream, and influence neural processes. Although the gut–brain axis is implicated in linking brain injury to gut microbial changes, the specific dynamics and mechanisms of this remain poorly elucidated.

Metabolites, as end products of biochemical reactions, directly reflect dynamic changes in cellular physiological activities. These small molecules participate in critical pathways such as energy metabolism and signal transduction, providing a vital window into the molecular mechanisms of biological processes [12]. Current research on mTBI and metabolomics has primarily focused on identifying biomarkers in liquid biospecimens including serum, cerebrospinal fluid, and urine, yielding promising results. For instance, after mTBI, blood levels of proteins such as Glial Fibrillary Acidic Protein (GFAP), Ubiquitin C-Terminal Hydrolase-L1 (UCH-L1), tau, and Neurofilament Light Chain (NfL) have been found to increase [13,14,15]. However, studies directly examining brain tissue at the injury site or peri-lesional areas in mTBI remain limited. Moreover, existing multi-omics research on mTBI has largely integrated microbiome and transcriptomic data, while the association between gut microbiota and brain metabolites in the repair mechanisms following mTBI remains largely unexplored.

In this study, we established an animal mTBI model to investigate microbial and metabolomic alterations. By collecting fecal samples (sham, 16 h, 1 d, 3 d, 5 d, 7 d, 14 d, and 2 m post-injury) and brain tissues (sham, 1 d, 3 d, 5 d, 7 d, 14 d post-injury), we performed a time-series multi-omics screening to capture the post-mTBI dynamics. This approach allowed us to preliminarily identify candidate biomarkers and elucidate the evolving correlations between the gut microbiome and brain metabolome throughout injury progression, providing initial mechanistic insights into gut–brain axis disruption.

## 2. Materials and Methods

### 2.1. Animals

All experimental animals were purchased from the Laboratory Animal Center of Central South University. To account for the potential confounding effects of estrogen, male Sprague-Dawley (SD) rats, with a weight range of 280–320 g, were procured from Hunan Silaikejingda Co., Ltd. and were housed in an animal facility under standard conditions (12/12 light–dark cycle, humidity at 60 ± 5%, temperature 23 ± 0.5 °C). During adaptation over 6 days, the rats were weighed every other day, until they had reached adulthood with a stabilized growth rate.

### 2.2. mTBI Model Establishment

The injury model was established as previously described [16]. Rats were deeply anesthetized with 3% isoflurane and placed on a destructible tin foil plane. A small metal disk (“helmet”) was positioned over the bregma to evenly distribute impact force. A wedge-shaped sponge (“pillow”) was placed under the neck to maintain the head and body in the same plane. A 550 g weight was dropped vertically from a height of 100 cm onto the helmet to induce mTBI. Upon impact, the tin foil ruptured, allowing the rat to fall and undergo a 180° rotation onto a foam pad, simulating the acceleration–deceleration forces commonly associated with concussion.

### 2.3. Sample Processing and Gut Preparation

Fecal samples were collected from eight groups: sham, 16 h (*n* = 3, as two samples were lost), 1 d, 3 d, 5 d (*n* = 4, as one sample was lost), 7 d, 14 d, and 2 m post-injury (*n* = 5 per group unless otherwise specified). The rats were stimulated to defecate with abdominal massage. Fresh fecal pellets were collected directly without contact with any external surfaces and immediately placed in 2 mL cryotubes. Samples were flash-frozen in liquid nitrogen and later stored at −80 °C until further processing.

Animals from six groups—sham, 1 d, 3 d, 5 d, 7 d, and 14 d post-injury (*n* = 6 per group)—were euthanized via cardiac perfusion under deep anesthesia induced by 3% isoflurane. The thoracic and abdominal cavities were opened to expose the heart. After perfusion, the rats were decapitated, and the whole brain was harvested. Half of the brain was dissected to isolate the hippocampus and the injured region (parietal cortex), which were placed in 1.2 mL cryotubes; the other hemisphere was stored in a cryotube as a backup. All brain samples were flash-frozen in liquid nitrogen and subsequently transferred to a −80 °C freezer for long-term storage. The proximal jejunum (approximately 6–8 cm in length) was dissected from the abdominal cavity. The upper segment was preserved in RNA stabilization solution for transcriptomic analysis, while the lower segment was fixed in 4% paraformaldehyde for H&E staining and immunofluorescence assays.

### 2.4. DNA Extraction and PCR Amplification

DNA was extracted using the OMEGA Soil DNA Kit (D5635-02) (Omega BioTek, Norcross, GA, USA) as per the manufacturer’s instructions. Further, DNA concentration and purity were verified using the NanoDrop 2000 (Thermo Scientific, Waltham, MA, USA) and 0.8% agarose gel electrophoresis.

The bacterial 16S rRNA V3–V4 hypervariable regions were amplified using the specific primers 338F (5′-ACTCCTACGGGAGGCAGCA-3′) and 806R (5′-GGACTACHVGGGTWTCTAAT-3′). The PCR mix for each sample included 5 μL of 5× Reaction Buffer, 5 μL of 5× High GC Buffer, 2 μL of 10 mM dNTPs, 1 μL of each primer, 0.25 μL of Q5 high-fidelity DNA polymerase, 2 μL of template DNA, and ddH_2_O to make up a final volume of 25 μL. The amplification protocol consisted of initial denaturation at 98 °C for 5 min; followed by 25 cycles of denaturation at 98 °C for 30 s, annealing at 53 °C for 30 s, and extension at 72 °C for 45 s; with a final extension at 72 °C for 5 min. The amplification products were visualized using 2% agarose gel electrophoresis, and quantification was performed using the Quant-iT™ PicoGreen™ dsDNA Assay Kit (Thermo Fisher Scientific, Waltham, MA, USA) on a microplate reader (BioTek Instruments, Winooski, VT, USA).

### 2.5. Illumina MiSeq Sequencing and Bioinformatics Analysis

The amplicons were purified, and a library was created by targeting the 16S V3 and V4 regions. Sequencing was performed using the Illumina MiSeq platform with a 300 bp paired-end library.

Bioinformatic processing of raw sequencing data was performed using QIIME2 software (version 2022.11), in which denoising, paired-end sequence merging, and chimera removal were performed using the DADA2 algorithm. The obtained Amplicon Sequence Variant (ASV) sequences were taxonomically annotated by alignment against the Greengenes 13_8 reference database. ASVs with a relative abundance below 0.001% of the total sequencing depth (considered low-abundance ASVs) were filtered out. ASV count comparisons and taxonomic difference analyses were visualized and conducted using R programming (version 4.2.2). The GenesCloud online platform (https://www.genescloud.cn/home (accessed on 26 January 2026) was used to perform alpha and beta diversity analyses. To identify significantly abundant taxa, linear discriminant analysis effect size (LEfSe) was performed with an LDA score > 2.

### 2.6. Untargeted Metabolome Analysis via Liquid Chromatography–Mass Spectrometry (LC–MS)

Parietal cortex tissue samples were weighed and homogenized with grinding beads at 30 Hz for 20 s; subsequently, 400 μL of 70% methanol containing internal standards was added, followed by oscillating extraction, incubation in ice, and centrifugation. Finally, 200 μL of the clarified supernatant was transferred to an injection vial for LC-MS analysis; more details of the LC-MS protocols are provided in Tables S1 and S2. Raw MS data (raw files) were converted to the mzXML format using ProteoWizard. Subsequent chromatographic peak detection, alignment, and feature extraction were performed using the XCMS algorithm. Metabolites with an annotation confidence score ≥ 0.5 were retained for subsequent analysis. Orthogonal Partial Least Squares Discriminant Analysis (OPLS-DA) was employed to screen for differentially abundant metabolites.

The variable importance in projection (VIP) value was obtained from each variable in the OPLS-DA model. Then, univariate analysis was performed to analyze the differences among the groups and to calculate the metabolites’ *p* values and FC values. The metabolites with VIP > 2, FDR < 0.05, and |log_2_(FC)| > 1 were considered significantly differentially abundant metabolites, and were subjected to pathway enrichment analysis based on the Kyoto Encyclopedia of Genes and Genomes (KEGG) database. To assess any associations between brain metabolites and gut microbiota, Pearson’s correlation coefficients were calculated.

### 2.7. Hematoxylin–Eosin (H&E) and Immunofluorescence Staining

The gut tissues harvested from each animal were immersion-fixed in a 4% paraformaldehyde solution for over 24 h. Following fixation, the tissues were processed through graded ethanol dehydration (75–100%), cleared in xylene, embedded in paraffin, and sectioned to a thickness of 3 μm. H&E staining was carried out utilizing an H&E staining kit (Wuhan Pinuofei Biotechnology, Wuhan, China) in strict adherence to the manufacturer’s protocols. The histopathology of the tissues was examined under a light microscope.

The gut sections underwent sequential deparaffinization in xylene (2 × 5 min) and rehydration through a graded ethanol series (100–75%). Antigen retrieval was performed through high-pressure heating of the sections in citrate buffer (pH 6.0) at 120 °C for 2 min; then, sections were blocked with 10% goat serum. Tissue sections were incubated overnight at 4 °C with the following primary antibodies: anti-ZO-1 (1:200), anti-Occludin (1:200), and anti-Claudin-1 (1:200). Following primary antibody incubation, the sections were incubated at room temperature with secondary antibody for 1 h and nuclei were stained with DAPI. Sections were coverslipped using an anti-fade mounting medium to preserve fluorescence signals during microscopy.

H&E and immunofluorescence sections were examined using SlideViewer and quantitatively analyzed using Image-Pro Plus 6.0. The mean fluorescence intensity of target regions was measured to evaluate the expression levels of target proteins. Data extracted from ImageJ were subjected to statistical analysis and graphing using GraphPad Prism version 9.0, and intergroup differences were assessed by one-way ANOVA, in which a *p*-value < 0.05 was considered statistically significant.

### 2.8. RNA Extraction and RT-qPCR

Total RNA was extracted from intestinal and brain tissues using the Trelief^®^ RNAprep FastPure Tissue & Cell Kit (Tsingke Biotechnology, Beijing, China), following the manufacturer’s instructions. Reverse transcription was performed with the SynScript^®^ III RT SuperMix for qPCR (+gDNA Remover, Tsingke Biotechnology), as per the manufacturer’s instructions. QPCR was carried out using the ArtiCanTM SYBR qPCR Mix (Tsingke Biotechnology) as directed. The 2^−ΔΔCt^ technique was used to examine the levels of messenger ribonucleic acid (mRNA) expression. Primer sequences were obtained from the NCBI GenBank database (https://www.ncbi.nlm.nih.gov (accessed on 26 January 2026) and verified for specificity using NCBI BLAST (https://blast.ncbi.nlm.nih.gov/Blast.cgi, accessed on 26 January 2026). All primers were synthesized by Tsingke Biotechnology Co., Ltd. (Beijing, China), the detailed sequences of which are provided in Table S3.

### 2.9. Statistical Analyses

Statistical analysis was performed using Graph Pad Prism 9.0. *p* < 0.05 was considered statistically significant. The associations between brain metabolomic profiles and gut microbial taxa were assessed using Pearson’s correlation analysis.

## 3. Results

### 3.1. Overview of 16S rRNA Gene Sequencing of the Gut Microbiota After mTBI

Fecal samples were collected from eight groups—controls, 16 h post-injury, 1 day post-injury, 3 days post-injury, 5 days post-injury, 7 days post-injury, 14 days post-injury, and 2 months post-injury (sham, 16 h, 1 d, 3 d, 5 d, 7 d, 14 d, and 2 m post-injury)—and a total of 37 specimens were successfully sequenced after quality control (Table S4). The sequence length of the obtained reads ranged from 230 bp to 442 bp across both the control and experimental groups, with the majority of fragments centered around 430 bp. After removing singleton sequences, the number of ASVs per sample ranged from 28,645 to 66,414. The sequencing quality was evaluated through rarefaction curves, species accumulation curves, and rank abundance curves. The rarefaction curve approached a plateau at its terminus (Figure S1a), indicating sufficient sequencing depth, and the species accumulation curve also reached a plateau in its late phase (Figure S1b), demonstrating adequate sample coverage. The rank abundance curve exhibited a generally gentle slope across most of its distribution (Figure S1c), suggesting a relatively uniform abundance among ASVs within the community.

**Figure 1:**
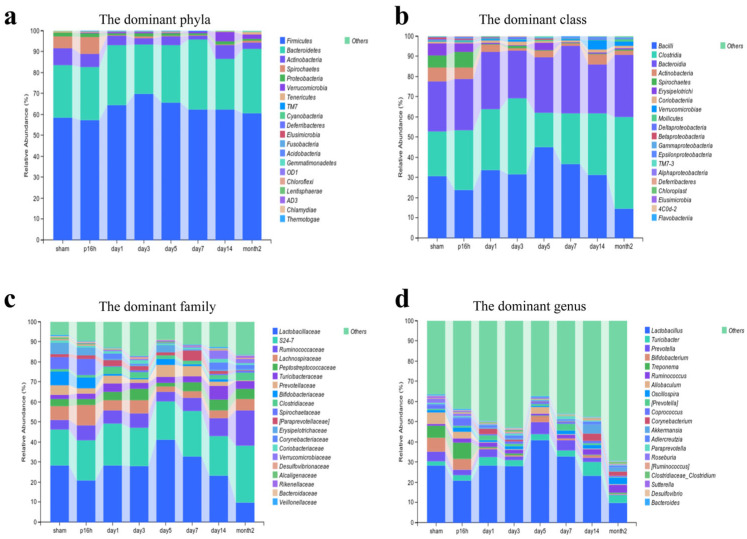
The dominant gut microbiota at different post-injury time points. (**a**) Bar plot of the percent of community abundance of the dominant phyla. (**b**) Bar plot of the percent of community abundance of the dominant class. (**c**) Bar plot of the percent of community abundance of the dominant family. (**d**) Bar plot of the percent of community abundance of the dominant genus. Sham, p16h, day1, day3, day5, day7, day14 and month2 correspond to fecal samples from controls, 16 h post-injury, 1 day post-injury, 3 days post-injury, 5 days post-injury, 7 days post-injury, 14 days post-injury, and 2 months post-injury, respectively.

The compositional distribution of the microbiota across the phylum, class, family, and genus levels was visualized based on a feature table with the singleton sequences removed. The dominant gut microbiota composition in the rats exhibited substantial variations after mTBI (Figure 1 and Figure S2). At the phylum level, the dominant taxa consisted primarily of *Firmicutes*, *Bacteroidota*, *Actinobacteria*, *Spirochaetes*, and *Proteobacteria* (Figure 1a); the most abundant classes were *Bacilli*, *Clostridia*, *Bacteroidia*, *Actinobacteria*, and *Spirochaetia* (Figure 1b); at the family level, *Lactobacillaceae*, *Ruminococcaceae*, *Lachnospiraceae*, and *Peptostreptococcaceae* were prevalent (Figure 1c); and among the top genera bacteria were *Lactobacillus*, *Turicibacter*, *Prevotella*, *Bifidobacterium*, and *Ruminococcus* (Figure 1d). The analysis of relative abundance trends among the top five taxa at the phylum, class, family, and genus levels demonstrated oscillatory alterations in the gut microbiota across all taxonomic tiers (Figure S2). Notably, *clostridia*, the second most dominant family, exhibited a fluctuating yet overall increasing trend post-injury, with a distinct trough observed on day 5 (Figure S2b). Conversely, both *Lactobacillacea* (the most dominant family) and *Lactobacillus* (the most dominant genus) exhibited a general decline in relative abundance, which paradoxically coincided with a transient peak on day 5 (Figure S2c,d). Other probiotic bacteria, such as *Lachnospiraceae* (Figure S2c) and *Bifidobacterium* (Figure S2d), also exhibited declining trends, although the changes were not statistically significant.

### 3.2. Temporal Profiling of Gut Microbiota Changes After mTBI

**Figure 2:**
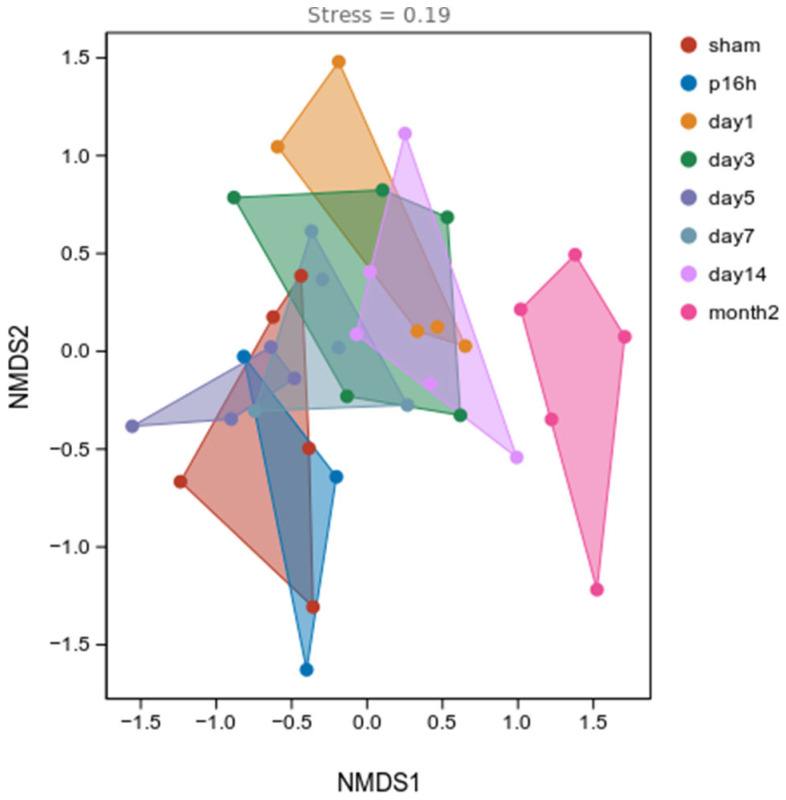
Non-metric multidimensional scaling (NMDS) with Bray–Curtis distance, showing the significant differences in the gut bacterial composition at different time points (stress: 0.19). Sham, p16, 1 d, 3 d, 5 d, 7 d, 14 d and month2 correspond to fecal samples from controls, 16 h post-injury, 1 day post-injury, 3 days post-injury, 5 days post-injury, 7 days post-injury, 14 days post-injury and 2 months post-injury, respectively.

To assess changes in the alpha diversity of gut microbiota in model rats at different post-injury time points, multiple ecological indices were employed, including the Chao1 index and Observed Species index (reflecting community richness) and the Shannon index and Simpson index (reflecting community diversity). Although no significant differences in alpha diversity indices were observed within the first 7 days post-injury compared to the sham group, both the Simpson and Shannon indices revealed a trend of initial decline followed by a recovery in gut microbiota diversity during the early phase after injury, with the lowest point reached at day 5 (Figure S3a). In contrast, a significant difference in gut microbiota alpha diversity was detected at 2 months post-injury (Figure S3b), which was more likely influenced by the extended time span. The distinction among groups was observed using non-metric multidimensional scaling (NMDS) with the Bray–Curtis distance, which showed a distinct change in the gut bacterial composition after mTBI (Figure 2).

A Permutational Multivariate Analysis of Variance (PERMANOVA) was performed to evaluate the differences among groups, with a larger pseudo-F statistic denoting greater separation (Table 1). The analysis demonstrated that all post-injury groups exhibited statistically significant distinctions from the sham group, providing further statistical evidence that mTBI induced significant shifts in the gut microbial community structure.

**Figure 3:**
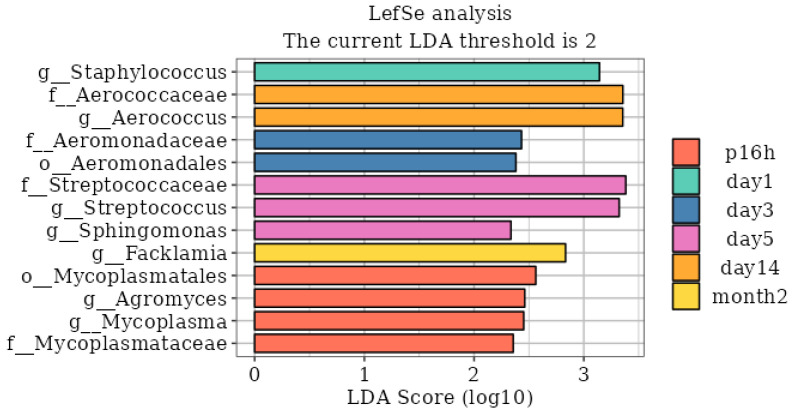
LEfSe analysis of gut microbiome at different time points. Sham, p16, 1 d, 3 d, 5 d, 14 d and Month2 correspond to fecal samples from controls, 16 h post-injury, 1 day post-injury, 3 days post-injury, 5 days post-injury, 14 days post-injury, and 2 months post-injury, respectively.

LEfSe analysis was performed with an LDA score > 2 to identify statistically significant biomarkers at different time points after injury (Figure 3). The results showed that at 16 h post-mTBI, the most significant gut microbiota were *Mycoplasma* and *Agromyces*; at 1 d post-mTBI, the most significant gut microbiota was *Staphylococcus*; at 3 d post-mTBI, *Aeromonadaceae* was the most abundant; at 5 d post-mTBI, *Streptococcus* and *Sphingomonas* were markedly enriched; by 14 d post-mTBI, *Aerococcus* showed significant differences; finally, at 2 months post-mTBI, *Facklamia* was determined to be statistically significant.

### 3.3. Characterization of the Global Metabolomic Landscape in the Brain After mTBI

**Figure 4:**
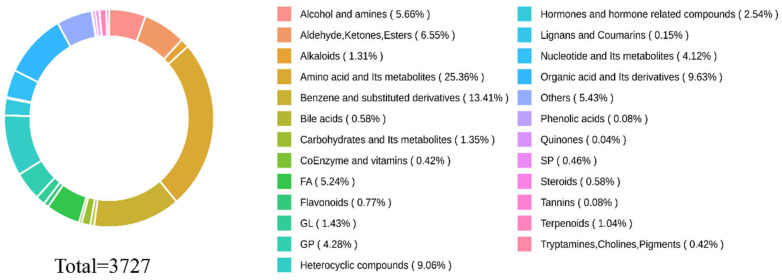
Composition of metabolites. The top 3 were amino acids and their metabolites (21.30%), benzene and substituted derivatives (13.79%), and organic acids and their derivatives.

A total of 36 parietal lobe tissue samples were included in this study. The untargeted metabolomic analysis identified 3727 metabolites, among which 2446 metabolites were detected in T3_positive mode and 1281 in T3_negative mode (Figure 4). Based on the HMDB and PubChem databases, these metabolites were categorized into 25 biochemical classes, including amino acids and their metabolites (25.36%); benzene and substituted derivatives (13.41%); organic acids and their derivatives (9.63%); heterocyclic compounds (9.06%); aldehydes, ketones, and esters (6.55%); fatty acyls (5.24%); alcohols and amines (5.66%); glycerophospholipids (4.28%); nucleotides and their metabolites (4.12%); hormones and hormone-related compounds (2.54%); glycolipids (1.43%); carbohydrates and their metabolites (1.35%); alkaloids (1.31%); terpenoids (1.04%); flavonoids (0.77%); bile acids (0.58%); steroids (0.58%); sphingolipids (0.46%); coenzymes and vitamins (0.42%); tryptamines, cholines, and pigments (0.42%); lignans and coumarins (0.15%); phenolic acids (0.08%); tannins (0.08%); quinones (0.04%); and others (5.43%). The most abundant components were amino acids and their metabolites, benzene and substituted derivatives, and organic acids and their derivatives.

The analysis of the differences in brain metabolic profiles between the mTBI groups at different time points (1 d, 3 d, 5 d, 7 d, 14 d) and the sham group using the OPLS-DA model revealed that although all of the mTBI groups showed separation from the sham group in the score plot, only the models for the 7-day and 14-day post-injury time points passed rigorous validation, as indicated by the high explanatory variance (R^2^Y) and cross-validated predictive ability (Q^2^) (Figure S4). Permutation testing confirmed the statistical significance of these models (*p* < 0.05).

The criteria of VIP > 2, FDR < 0.05, and |log_2_(FC)| > 1 were applied to identify differential metabolites. The analysis revealed that at 1 d post-injury, only one metabolite was significantly increased; at 3 d, two were increased; at 5 d, one was increased; at 7 d, four were increased; and at 14 d, eight were increased and seven decreased (Table 2), which were primarily categorized as glycerophospholipids (90), organic acids and derivatives (21), and benzene and derivatives (19), among others, according to the KEGG database (Figure S5).

The most significantly up-/downregulated metabolites at each time point were 6-[4-(3-{3-[6-carboxy-5-(2,4-dihydroxyphenyl)-3-methylcyclohex-2-en-1-yl]-2,4-dihydroxyphenyl}-3-oxopropyl)-3-hydroxyphenoxy]-3,4,5-trihydroxyoxane-2-carboxylic acid on day 1 (up); PA(18:1(9Z)/22:5(4Z,7Z,10Z,13Z,16Z)) on day 3 (up); 1-hexadecanoyl-2-(9Z,12Z-octadecadienoyl)-sn-glycero-3-phosphoethanolamine on day 5 (up); PA(18:2(9Z,12Z)/24:1(15Z)) on day 7 (up); C24:1 Sphingomyelin (down); and Thioetheramide PC on day 14 (up). In addition, 1-hexadecanoyl-2-(9Z,12Z-octadecadienoyl)-sn-glycero-3-phosphoethanolamine, PA(18:2(9Z,12Z)/24:1(15Z)), and PC(16:0/20:4(5Z,8Z,11Z,14Z)) showed significant differences at multiple time points, respectively.

### 3.4. KEGG Enrichment Analysis of Differential Metabolites

**Figure 5:**
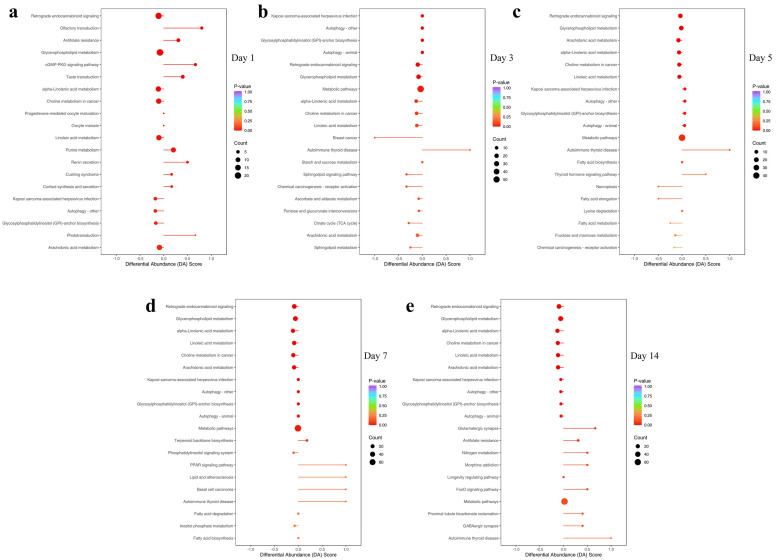
KEGG enrichment pathway diagram. (**a**–**e**) show the top 20 most significantly enriched pathways at 1 day post-injury, 3 days post-injury, 5 days post-injury, 7 days post-injury, and 14 days post-injury, respectively. The color of each dot represents the *p*-value and the size of each dot represents the number of significantly differential metabolites enriched in the corresponding pathway.

The KEGG pathways significantly enriched by the differential metabolites after mTBI mainly included glycerophospholipid metabolism (ko00564), retrograde endocannabinoid signaling (ko04723), linoleic acid metabolism (ko00591), α-Linolenic acid metabolism (ko00592) and so on (Figure 5). These findings indicate that the metabolic alterations after mTBI primarily involve the pathways mentioned above, among which glycerophospholipid metabolism and retrograde endocannabinoid signaling were the most significantly affected.

### 3.5. Integrated Analysis of the Microbiome and Metabolome

To investigate the correlation between brain injury and gut alterations, Spearman’s rank correlation analysis was performed on the gut microbiota and brain metabolome across various taxonomic levels. A heatmap was constructed to visualize the correlations between the top 20 brain metabolites and bacterial species at the genus level. The red and blue ellipses indicate positive and negative correlations (*p* < 0.05), respectively, with the ellipse narrowness representing the absolute correlation strength. The results revealed time-dependent alterations in the microbiota–metabolite correlations after injury (Figure S6).

At 1 d post-injury, *Adlercreutzia* and *Corynebacterium* exhibited significant positive correlations with L-Homophenylalanine and PA (18:1(9Z)/22:5(4Z,7Z,10Z,13Z,16Z)), but negative correlations with PE-NMe(14:0/15:0) and PC (14:0/22:4(7Z,10Z,13Z,16Z)) (Figure S6a). By 3 d, *Allobaculum*, *Bifidobacterium*, and *Sutterella* showed significant positive correlations with C24:1 Sphingomyelin, Colfosceril Palmitate, and PE (18:0/20:4(5Z,8Z,11Z,14Z)), while correlating negatively with 2′-Norberbamunine (Figure S6b). At 5 d, *Streptococcus* was positively correlated with 1-hexadecanoyl-2-(11Z-octadecenoyl)-sn-glycero-3-phosphothanolamine and Liothyronine, but negatively correlated with C24:1 Sphingomyelin and Colfosceril Palmitate (Figure S6c). At 7 d, the microbial–metabolomic associations began to shift. *Allobaculum* and *Sutterella* were positively correlated with PE-NMe(15:0/18:1(9z)), but negatively correlated with 1-hexadecanoyl-2-(11Z-octadecenoyl)-sn-glycero-3-phosphothanolamine, which, conversely, showed correlations with *Prevotella*. Notably, Thioetheramide PC demonstrated significant microbiota correlations for the first time at this stage, showing a positive correlation with *Prevotella* and negative correlations with *Sutterella* and *Allobaculum*, suggesting that the microbiota may progressively regulate specific lipid metabolic pathways during the chronic phase (Figure S6d). By 14 d, *Helicobacter*, *Adlercreutzia*, and *Corynebacterium* showed positive correlations with Thioetheramide PC (Figure S6e).

Further analysis revealed that the gut microbiota *Bifidobacterium* and *Sutterella* exhibited significant associations with brain metabolites at multiple time points post-injury, whereas *Streptococcus* showed significant correlations only after a specific time point (5 d). Similarly, the brain metabolites C24:1 Sphingomyelin, Colfosceril Palmitate, and Liothyronine were significantly correlated with the gut microbiota across multiple time points following injury, while Thioetheramide PC displayed significant associations only after a specific time point (7 d).

**Figure 6:**
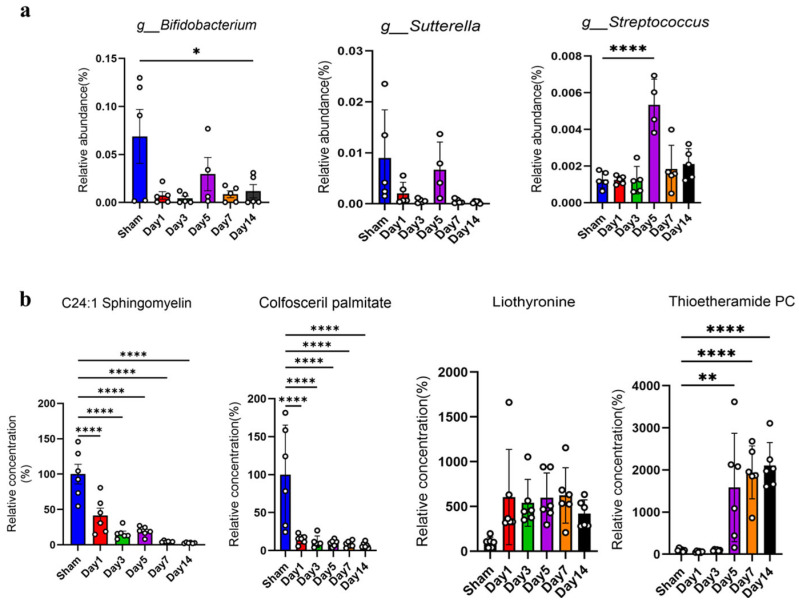
Changes in selected gut microbial abundances and brain metabolite levels after mTBI. (**a**). Changes in gut microbial relative abundances after mTBI. (**b**). Changes in relative brain metabolite levels after mTBI. Data were presented as mean ± SEM, with *n* = 5 per group in Figure 6a (one sample was lost at Day 5) and *n* = 6 per group in Figure 6b. * *p* < 0.05, ** *p* < 0.01, **** *p* < 0.0001 (one-way ANOVA with Dunnett’s multiple comparisons test).

The abundances of the aforementioned gut microbiota and the concentrations of the brain metabolites were quantified. The results showed that the *Bifidobacterium* and *Sutterella* abundances exhibited an overall decreasing trend after mTBI, with transient recovery observed on day 5 (Figure 6a); in contrast, *Streptococcus* showed a significant increase in abundance on day 5 (Figure 6a). Regarding the brain metabolites, C24:1 Sphingomyelin and Colfosceril Palmitate showed a sustained significant decrease after mTBI, whereas Liothyronine displayed a continuous increasing trend (although this did not reach statistical significance) (Figure 6b). Thioetheramide PC, however, exhibited a significant increase in concentration after day 5 (Figure 6b).

In summary, the acute phase (1 d–5 d) post-injury was predominantly characterized by significant associations between the gut microbiota and amino acid, phospholipid, reflecting a rapid microbial adaptation to the mTBI environment. By 14 d, the interplay between phospholipid metabolism and the gut microbiota became more pronounced, underscoring the long-term impact of the microbiota on lipid metabolism. Day 5 may represent a pivotal turning point in the dynamics of the gut microbiota and brain metabolites, with *Streptococcus* and Thioetheramide PC implicated as key players.

### 3.6. Inflammatory Factors May Mediate Brain–Gut Crosstalk After mTBI

Intestinal tissue samples were collected from the sham group and at 1, 3, 5, 7, and 14 days post-mTBI for H&E staining (*n* = 2 per group), observations which revealed that both the sham and mTBI groups maintained relatively good integrity of the jejunal mucosa, with no obvious signs of ulceration or loss of mucosal integrity (Figure S7). In the sham group, the small intestinal villi were neatly arranged; the columnar cells exhibited elongated oval nuclei located near the base of the cells; goblet cells, resembling wine glasses in shape, were scattered among the columnar cells, with their apices filled with mucigen granules; and scattered lymphocytes were also observed. After mTBI, intestinal mucosal atrophy was evident, and an increased number of neutrophils with deeply stained, curved rod-shaped or lobulated nuclei visible between the cells. By day 7 post-mTBI, swelling, shedding, and a reduced number of small intestinal villi were observed.

**Figure 7:**
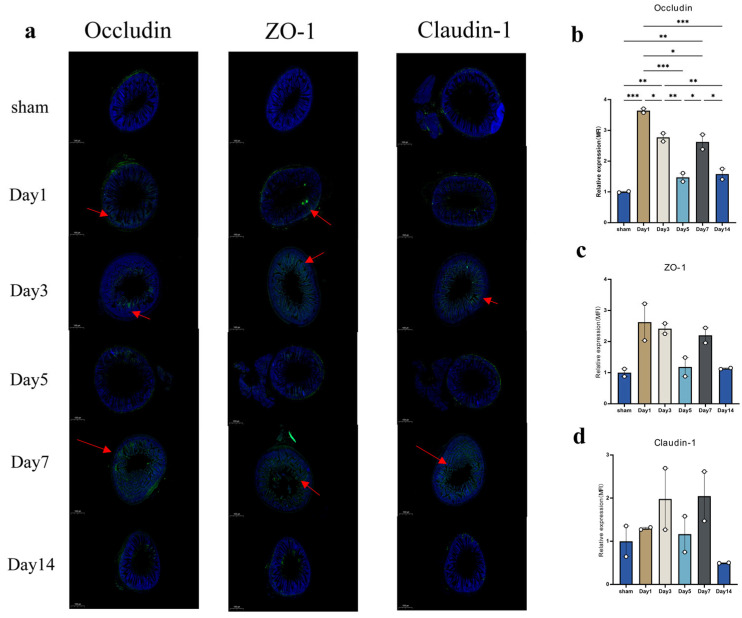
Impairment of the intestinal barrier after mTBI. (**a**) shows immunofluorescence staining of intestinal tissues at various time points after mTBI. Nuclei are counterstained with DAPI (blue); tight junction proteins (Occludin, ZO-1, and Claudin-1) appear in green. (**b**–**d**) show the quantitative analysis of immunofluorescence intensity for Occludin, ZO-1, and Claudin-1, respectively. Data were presented as mean ± SEM, with *n* = 2 per group. * *p* < 0.05, ** *p* < 0.01, *** *p* < 0.001 (one-way ANOVA with Tukey’s test).

Immunofluorescence staining was performed on intestinal tissues using antibodies against Occludin, ZO-1, and Claudin-1 to assess the expression levels of tight junction proteins at different time points after mTBI (Figure 7a). The results showed a consistent temporal pattern in the expression of all three proteins, with day 5 appearing to represent a turning point. This pattern likely reflects the dynamic changes in the intestinal barrier post-injury, including an initial phase of barrier repair (days 1–3), followed by mid-phase impairment (5 d), subsequent partial recovery (7 d), and a final phase suggestive of either barrier remodeling or incomplete repair (14 d). Among these proteins, the changes in Occludin expression reached statistical significance (Figure 7b), whereas the alterations in ZO-1 and Claudin-1 expression, despite showing certain trends, did not reach statistical significance (Figure 7c,d).

**Figure 8:**
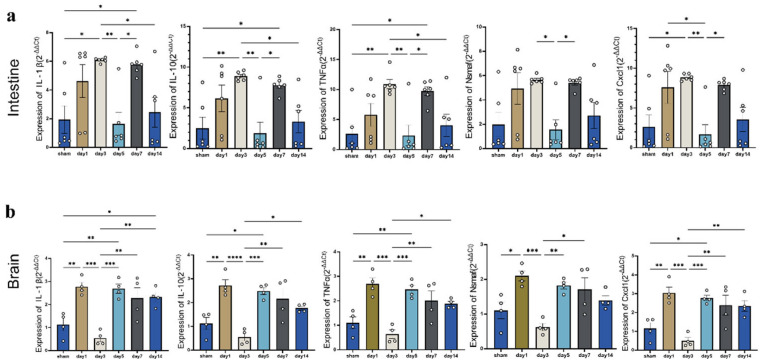
Expression levels of inflammatory factors in the intestine and brain. (**a**). The expression levels of inflammatory factors in the intestinal tissue. (**b**). The expression levels of inflammatory factors in the brain tissue. Data were presented as mean ± SEM, with *n* = 6 per group in Figure 8a and *n* = 4 per group in Figure 8b. * *p* < 0.05, ** *p* < 0.01, *** *p* < 0.001, **** *p* < 0.0001 (one-way ANOVA with Tukey’s test).

Alterations in intestinal permeability are often associated with the onset of intestinal inflammation. In line with findings from the microbiome and metabolome analyses, the expression of IL-1β, IL-10, TNF-α, Nsmaf (Neutral Sphingomyelinase Activation Associated Factor), and Cxcl1 was measured in the rat intestinal and brain tissues (*n* = 6 per group, as two brain sample was lost), which play crucial roles in mediating pro- and anti-inflammatory responses (Figure 8).

A congruent trend in the expression dynamics of the five cytokines was observed in both the intestinal and brain tissues. In the intestine, cytokine levels increased rapidly at 1 to 3 days post-injury, briefly declined at 5 d, rose again at 7 d, and then gradually decreased by 14 d, albeit remaining higher than those in the sham group (Figure 8a). In the brain tissue, cytokine levels surged immediately at day 1 post-injury, experienced a transient decrease at 3 d, and subsequently maintained elevated levels from 5 d to 14 d (Figure 8b). Notably, the changes in intestinal cytokines appeared to lag behind those observed in the brain.

## 4. Discussion

Mild Traumatic Brain Injury (mTBI) is one of the most common types of head injuries encountered in clinical and forensic practice [17]. Given its subtle pathological severity and nonspecific clinical manifestations, its forensic identification remains challenging. Previous studies have demonstrated that TBI can alter the gut microbiota, with significant shifts in microbial diversity and relative abundance observed within the short term (e.g., 24 h) post-injury [18]. Our earlier work further confirmed notable changes in the relative abundance of gut microbiota at both the phylum and family levels following mTBI [19]. However, the specific microbial taxa that may serve as forensically applicable biomarkers for mTBI are yet to be conclusively determined. Consequently, this study aims to systematically investigate the dynamic alterations in the gut microbiota after mTBI, with the objective of identifying potential biomarkers suitable for forensic practice. Furthermore, we analyzed the temporal dynamics of brain metabolites post-mTBI and performed an integrative analysis of gut microbiota and cerebral metabolic profiles to explore the molecular-level interactions within the gut–brain axis following mTBI.

Our findings demonstrated that the gut microbiota exhibited significant, multi-level dysbiosis across phylogenetic hierarchies (from phylum to genus) at various time points following mTBI, a pattern consistent with observations reported in a weight-drop mTBI model involving craniectomy [19]. The data revealed that *Lactobacillaceae*, the most abundant beneficial family, and *Lactobacillus*, the most abundant beneficial genus, displayed a similar fluctuating yet overall declining trend after injury, with a transient peak observed on day 5. Other beneficial taxa, including *Lachnospiraceae* and *Bifidobacterium*, also showed decreasing tendencies, although these changes did not reach statistical significance. These beneficial microbial groups are known to promote the production of short-chain fatty acids (SCFAs), particularly butyrate, which enhances the expression of tight junction proteins (such as ZO-1, Claudin-1, and Occludin) in vascular endothelial cells. This mechanism contributes to maintaining the integrity of the intestinal and blood–brain barriers, ultimately influencing the repair process after brain injury [20,21,22,23].

The LEfSe analysis identified eight significantly different microbial taxa. *Staphylococcus*, the most significant gut microbiota at 1 d post-mTBI, can induce host secretion of cytokines and chemokines (TNF-α, IL-1β, IL-10, and granulocyte colony-stimulating factor) [24,25,26]. Similarly, *Aeromonadaceae*, which was the most obvious at 3 d post-mTBI, can also induce the production of TNFα and IL-1β [27]. Furthermore, *Streptococcus*, markedly enriched at 5 d post-mTBI, has been shown to significantly elevate serum levels of pro-inflammatory factors such as IL-1β, IL-2, IL-6, and TNF-α in mice [28]. These differential gut microbiotas may be useful in estimating the post-injury time interval and assessing the cerebral and intestinal immune microenvironment following mTBI.

Brain metabolites serve as direct indicators of the biological processes of injury and repair following mTBI. Integrating microbiome data with metabolomic profiles may contribute to a better understanding of the complex bidirectional gut–brain regulatory mechanisms post-mTBI. Across different time points after injury, we identified a total of 17 differential metabolites, which were primarily glycerophospholipids, consistent with findings from a diagnostic study involving adolescent hockey players [29]. The KEGG pathway analysis confirmed significant disturbances in glycerophospholipid metabolism and retrograde endocannabinoid signaling. Given that lipids constitute a major component of brain tissue, the changes in glycerophospholipids likely reflect injury-induced inflammatory responses and increased demand for membrane repair [30,31]. Concurrently, the enrichment of endocannabinoid signaling may indicate activated neuroprotection, as molecules like 2-arachidonoyl glycerol (2-AG), N-arachidonoylserine (AraS), and N-palmitoylserine (PalmS) have demonstrated benefits in TBI models by reducing edema, neuronal loss, and neuroinflammation [32,33,34,35]. Thus, these metabolic shifts may represent both pathological responses and crucial recovery mechanisms post-mTBI.

Several gut microbiota and brain metabolites exhibited strong correlations at different time points following mTBI. Notably, *Streptococcus* was identified as a differentially abundant gut microbiota on day 5, while Thioetheramide PC and C24:1 Sphingomyelin emerged as the most significantly up-/downregulated differential metabolites, respectively, on day 14. An analysis of the temporal changes in these strongly correlated gut microbiota and brain metabolites revealed that *Streptococcus* showed a significant increase in abundance on day 5, Thioetheramide PC exhibited a marked rise in concentration after day 5, and C24:1 Sphingomyelin demonstrated a sustained and significant decrease after mTBI.

C24:1 sphingomyelin is a major component of myelin, essential for nerve conduction [36,37]. Aberrant sphingomyelin metabolism, including the impaired activity of sphingomyelinase leading to sphingomyelin accumulation, is documented in neurodegenerative diseases like Alzheimer’s disease, where it is linked to Aβ production and can manifest as neurological impairments such as dementia [38,39]. Thioetheramide PC competitively inhibits phosphatidylcholine hydrolysis by secretory phospholipase A2 (sPLA2), which plays a complex and critical dual role in brain physiology and pathology. After brain injury, sPLA2 is highly activated, producing abundant arachidonic acid and leading to various neural damage including neuroinflammation and oxidative stress [40,41,42]. The sustained decrease in C24:1 sphingomyelin suggests prolonged cerebral immune–inflammatory activity and neural damage after injury. In contrast, the persistent upregulation of Thioetheramide PC reflects endogenous repair mechanisms, and the delayed elevation of Thioetheramide PC beyond day 5 may result from prior sPLA2 overactivation triggering a subsequent inhibitory feedback response. Based on the temporal analysis of differential gut microbiota and metabolites, day 5 post-injury is proposed as a key time point in mTBI progression.

Immune mechanisms mediated through the gut–brain axis may explain the associations observed between gut microbiota and brain metabolites. Studies have indicated that TBI can lead to an increase in NF-κB within the gut, triggering an acute inflammatory response that reduces the expression of tight junction proteins and promotes apoptosis of intestinal epithelial cells, thereby increasing intestinal permeability and disrupting gut microbiota homeostasis [43,44,45,46]. Furthermore, gut microbiota can directly or indirectly influence the cerebral immune environment by mediating the migration of gut immune cells to the brain and regulating the secretion of gut-derived metabolites [47]. In this study, the expression levels of intestinal tight junction proteins (ZO-1, Occludin, and Claudin-1) and intestinal inflammatory factors exhibited consistent fluctuating changes after mTBI, reaching their lowest point on day 5. In contrast, the lowest expression levels of brain inflammatory factors occurred earlier, on day 3, which suggests that alterations in the intestinal immune environment following mTBI may lag behind those in the brain. How bidirectional immune regulation along the gut–brain axis links gut microbiota with brain metabolites requires further investigation and validation.

Nevertheless, this study has several limitations. In terms of animal model, the exclusive use of male rats limits the generalizability of the findings to other sexes and age group, which should be a focus of future research. Concerning study design, the identified candidate biomarkers and correlations require rigorous experimental and statistical validation. Furthermore, the observational and correlative nature of the design precludes causal inference; further interventional studies (e.g., microbial transplantation, metabolite supplementation) are needed to confirm the proposed gut–brain links. Regarding methodology, the lack of functional behavioral assessments weakens the connection between gut alterations and neurological outcomes. Additional specific staining for brain immune cells and myelin integrity would provide more direct histological support for the reported neuroinflammation and myelin disruption. Moreover, the immunofluorescence analysis of tight junction proteins (Occludin, ZO-1, Claudin-1) requires refinement to accurately determine their precise alterations.

## 5. Conclusions

This study reveals coordinated, time-dependent disruptions in the gut–brain axis after mTBI, proposing novel biomarker candidates for forensic investigation. Specifically: *Staphylococcus*, *Aeromonadaceae* and *Streptococcus* are markedly enriched at 1 d, 3 d and 5 d post-mTBI, respectively. Altered brain metabolites correlate temporally with specific microbial shifts. Gut immune changes lag behind central responses, suggesting a sequential dialogue. Collectively, this work provides a new direction for research into the forensic diagnosis and mechanistic underpinnings of mTBI.

## Figures and Tables

**Table 1:** Results of PERMANOVA based on Bray–Curtis distance.

Group1	Group2	Sample Size	Permutations	Pseudo-F	*p*-Value	q-Value
all	-	37	999	2.325	0.001	-
sham	p16h	8	999	0.515	0.844	0.844
sham	1 d	10	999	2.765	0.009	0.025
sham	3 d	10	999	2.022	0.016	0.032
sham	5 d	9	999	2.141	0.044	0.062
sham	7 d	10	999	2.176	0.015	0.032
sham	14 d	10	999	2.738	0.007	0.025
sham	month2	10	999	3.691	0.008	0.025
p16h	1 d	8	999	2.156	0.02	0.037
p16h	3 d	8	999	1.790	0.044	0.062
p16h	5 d	7	999	2.165	0.064	0.085
p16h	7 d	8	999	2.263	0.037	0.058
p16h	14 d	8	999	2.402	0.015	0.032
p16h	month2	8	999	2.861	0.025	0.041
1 d	3 d	10	999	0.672	0.835	0.844
1 d	5 d	9	999	4.014	0.011	0.028
1 d	7 d	10	999	1.506	0.089	0.113
1 d	14 d	10	999	1.396	0.134	0.163
1 d	month2	10	999	2.660	0.007	0.025
3 d	5 d	9	999	2.707	0.021	0.037
3 d	7 d	10	999	0.873	0.502	0.541
3 d	14 d	10	999	0.986	0.464	0.520
3 d	month2	10	999	2.632	0.006	0.025
5 d	7 d	9	999	2.458	0.005	0.025
5 d	14 d	9	999	3.650	0.004	0.025
5 d	month2	9	999	4.982	0.008	0.025
7 d	14 d	10	999	1.436	0.148	0.173
7 d	month2	10	999	3.788	0.009	0.025
14 d	month2	10	999	2.518	0.007	0.025

* “Group 1” and “Group 2” indicated the two groups being compared. “Sample size” denoted the sum of samples included in the comparison.

**Table 2:** Changes in brain metabolites after mTBI.

Groups	Metabolites	VIP	FDR	Log_2_FC	Up/Down
Day1	**6-[4-(3-{3-[6-carboxy-5-(2,4-dihydroxyphenyl)-3-methylcyclohex-2-en-1-yl]-2,4-dihydroxyphenyl}-3-oxopropyl)-3-hydroxyphenoxy]-3,4,5-trihydroxyoxane-2-carboxylic acid**	2.789	0.006	2.447	up
Day3	**PA(18:1(9Z)/22:5(4Z,7Z,10Z,13Z,16Z))**	2.712	0.020	1.392	up
	1-hexadecanoyl-2-(9Z,12Z-octadecadienoyl)-sn-glycero-3-phosphoethanolamine	2.716	0.001	1.141	up
Day5	**1-hexadecanoyl-2-(9Z,12Z-octadecadienoyl)-sn-glycero-3-phosphoethanolamine**	3.123	<0.001	1.151	up
Day7	1-hexadecanoyl-2-octadecanoyl-sn-glycero-3-phosphocholine	2.622	0.006	2.529	up
	**PA(18:2(9Z,12Z)/24:1(15Z))**	2.642	0.049	3.534	up
	PC(16:0/20:4(5Z,8Z,11Z,14Z))	2.667	0.001	1.835	up
	1-hexadecanoyl-2-(9Z,12Z-octadecadienoyl)-sn-glycero-3-phosphoethanolamine	2.633	0.003	0.998	up
Day14	**C24:1 Sphingomyelin**	2.831	0.040	(5.183)	down
	6beta-Naltrexol	2.268	0.029	1.107	up
	3-Oxo-octanoic acid (2-oxo-tetrahydro-furan-3-YL)-amide	2.251	0.031	1.402	up
	**Thioetheramide PC**	2.792	0.024	4.396	up
	1-eicosanoyl-2-(9Z-tetradecenoyl)-glycero-3-phosphocholine	2.704	0.029	2.551	up
	Tyr-Val-Lys-Ala-Leu	2.649	0.011	(1.075)	down
	PE-NMe2(18:1(9Z)/18:1(9Z))	2.641	0.032	(2.746)	down
	LPC(0:0/20:5)	2.587	0.003	(1.214)	down
	PA(18:2(9Z,12Z)/24:1(15Z))	2.704	0.011	3.405	up
	Ser-Pro-Thr-Phe-Leu	2.695	0.007	(1.260)	down
	NCGC00386087-01_C43H60N2O12_(2S)-N-[(2E,4E,6S,7R)-7-{(3S,4R)-3,4-Dihydroxy-5-[(1E,3E,5E)-7-(4-hydroxy-2-oxo-1,2-dihydro-3-pyridinyl)-6-methyl-7-oxo-1,3,5-heptatrien-1-yl]tetrahydro-2-furanyl}-6-methoxy-5-methyl-2,4-octadien-1-yl]-2-{(2R,3R,4R,6S)-2,3,4-trihydroxy-5,5-dimethyl-6-[(1E,3E)-1,3-pentadien-1-yl]tetrahydro-2H-pyran-2-yl}butanamide	2.235	0.038	1.994	up
	1-Stearoyl-2-oleoyl-sn-glycero-3-phosphocholine	2.778	0.001	(1.68)	down
	PC(16:0/20:4(5Z,8Z,11Z,14Z))	2.716	0.002	1.94	up
	1-hexadecanoyl-2-(9Z,12Z-octadecadienoyl)-sn-glycero-3-phosphoethanolamine	2.70	0.002	1.09	up
	6-({8-[3,7-dihydroxy-2-(3-hydroxyphenyl)-3,4-dihydro-2H-1-benzopyran-4-yl]-3,5-dihydroxy-2-(3-hydroxyphenyl)-3,4-dihydro-2H-1-benzopyran-7-yl}oxy)-3,4,5-trihydroxyoxane-2-carboxylic acid	2.61	0.029	(1.78)	down

* Sham, p16, 1 d, 3 d, 5 d, 7 d, 14 d, and Month2 correspond to fecal samples from controls, 16 h post-injury, 1 day post-injury, 3 days post-injury, 5 days post-injury, 7 days post-injury, 14 days post-injury, and 2 months post-injury, respectively. The most significantly upregulated or downregulated metabolites at each time point are shown in bold. The screening criteria were defined as VIP > 2, FDR < 0.05, and |log_2_(FC)| > 1.

## Data Availability

The original contributions presented in this study are included in the article/Supplementary Materials. Further inquiries can be directed to the corresponding authors.

## Supplementary Materials

The following supporting information can be downloaded at https://www.mdpi.com/article/10.3390/biomedicines14020311/s1, Figure S1: The sequencing quality evaluated through rarefaction curves, species accumulation curves, and rank abundance curves. Figure S2: The relative abundance trends among the top five taxa at the phylum, class, family, and genus levels. Figure S3: The alpha diversity of gut microbiota in model rats at different post-injury time points. Figure S4: Scores of OPLS-DA plot at different post-injury time points. Figure S5: Classification plot of significantly differential metabolites. Figure S6: Heatmap of correlations between the top 20 differential metabolites and microorganisms. Figure S7: H&E staining of intestinal tissues at various time points after mTBI under 10×, 20×, and 40× magnification. Table S1: Conditions of liquid chromatography. Table S2: Conditions of AB TripleTOF6600 mass spectrometry. Table S3: Primer Sequences in RT-qPCR. Table S4: Sequencing yield of gut microbiota samples in weight-drop model.

## Abbreviations

The following abbreviations are used in this manuscript:

mTBI	Mild Traumatic Brain Injury
SD	Sprague-Dawley
AD	Alzheimer’s Disease
PD	Parkinson’s Disease
TBI	Traumatic Brain Injury
CNS	Central Nervous System
FMT	Fecal Microbiota Transplantation
GFAP	Glial Fibrillary Acidic Protein
UCH-L1	Ubiquitin C-Terminal Hydrolase-L1
NfL	Neurofilament Light Chain
ASV	Amplicon Sequence Variant
LEfSe	Linear Discriminant Analysis Effect Size
LC–MS	Liquid Chromatography–Mass Spectrometry
OPLS-DA	Orthogonal Partial Least Squares-Discriminant Analysis
VIP	Variable Importance
NMDS	Non-Metric Multidimensional Scaling
PERMANOVA	Permutational Multivariate Analysis of Variance
Nsmaf	Neutral Sphingomyelinase Activation Associated Factor
SCFA	Short-Chain Fatty Acid
2-AG	2-Arachidonoyl Glycerol
AraS	N-arachidonoylserine
PalmS	N-palmitoylserine
sPLA2	Secretory Phospholipase A2
