# Supplementary material for: Multi-Omics Profiling of mTBI-Induced Gut–Brain Axis Disruption: A Preliminary Study for Biomarker Screening and Mechanistic Exploration
Source: Biomedicines. 2026 Jan 30;14(2):311. doi: 10.3390/biomedicines14020311 (PMC12938811; doi:10.3390/biomedicines14020311)
Supplement: Supplementary file 1 [file biomedicines-14-00311-s001.zip › Supplementary Figures.zip!/Fig S1.pdf]

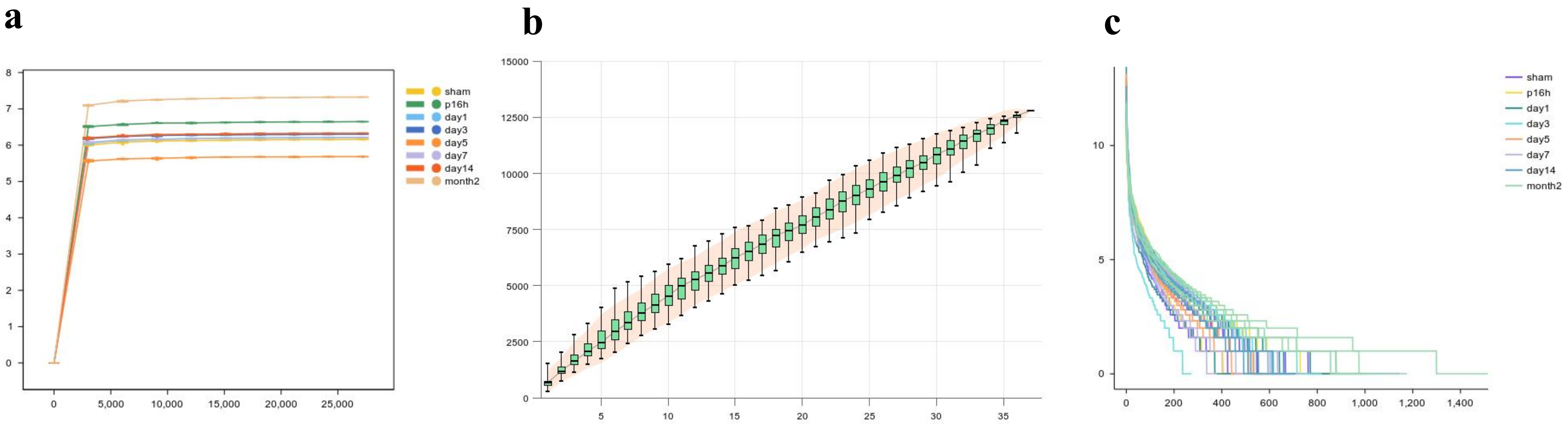
a
b
c
sham
p16h
day1
day3
day5
day7
day14
month2
0
5,000
10,000
15,000
20,000
25,000
15000
12500
10000
7500
5000
2500
0
5
10
15
20
25
30
35
200
400
600
800
1,000
1,200
1,400
