# Supplementary material for: Multi-Omics Profiling of mTBI-Induced Gut–Brain Axis Disruption: A Preliminary Study for Biomarker Screening and Mechanistic Exploration
Source: Biomedicines. 2026 Jan 30;14(2):311. doi: 10.3390/biomedicines14020311 (PMC12938811; doi:10.3390/biomedicines14020311)
Supplement: Supplementary file 1 [file biomedicines-14-00311-s001.zip › Supplementary Figures.zip!/Fig S2.pdf]

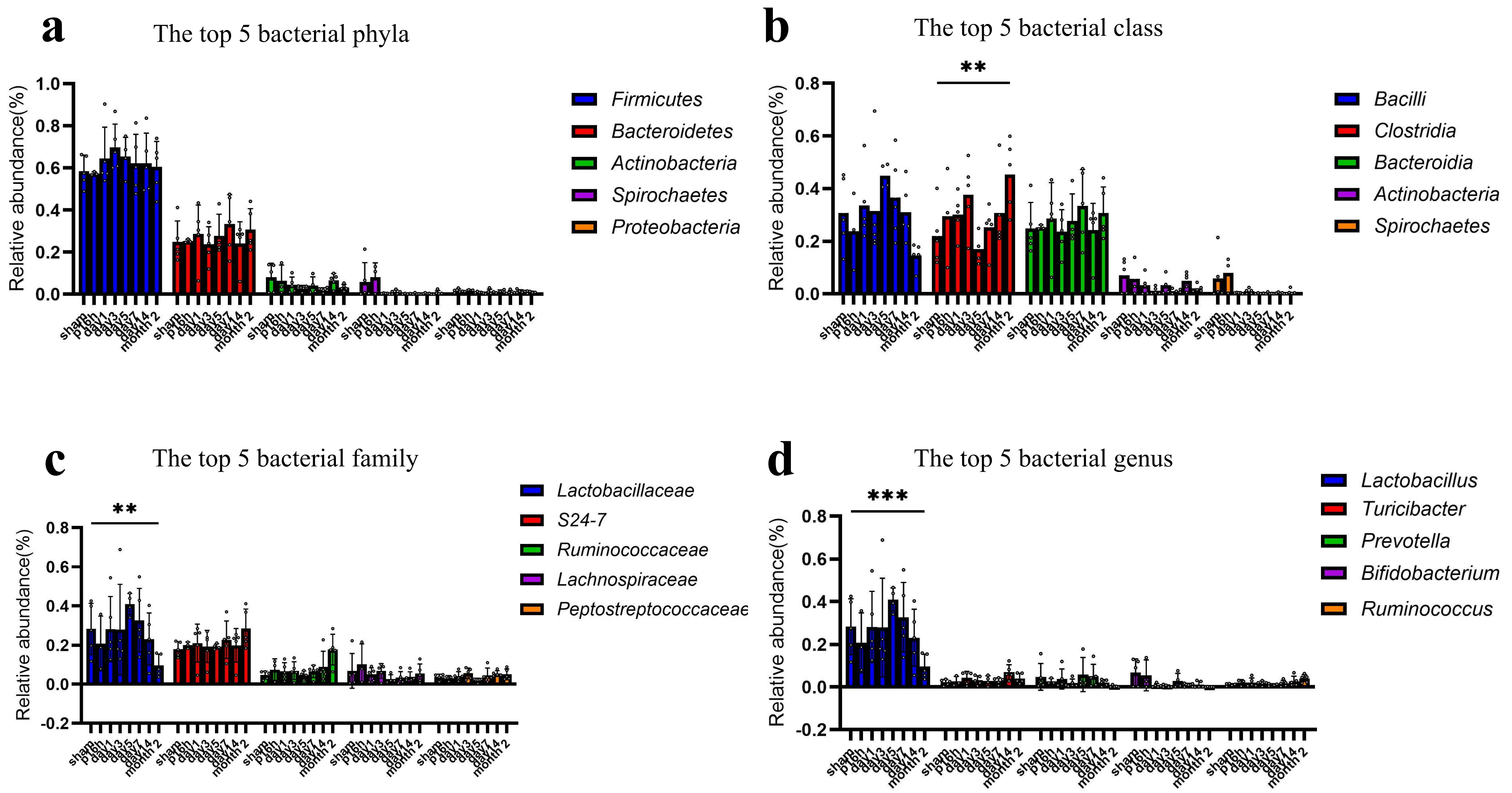
a
The top 5 bacterial phyla
Relative abundance(%)
Firmicutes
Bacteroidetes
Actinobacteria
Spirochaetes
Proteobacteria
b
The top 5 bacterial class
**
Relative abundance(%)
Bacilli
Clostridia
Bacteroidia
Actinobacteria
Spirochaetes
c
The top 5 bacterial family
**
Relative abundance(%)
Lactobacillaceae
S24-7
Ruminococcaceae
Lachnospiraceae
Peptostreptococcaceae
d
The top 5 bacterial genus
***
Relative abundance(%)
Lactobacillus
Turicibacter
Prevotella
Bifidobacterium
Ruminococcus
