# Supplementary material for: Multi-Omics Profiling of mTBI-Induced Gut–Brain Axis Disruption: A Preliminary Study for Biomarker Screening and Mechanistic Exploration
Source: Biomedicines. 2026 Jan 30;14(2):311. doi: 10.3390/biomedicines14020311 (PMC12938811; doi:10.3390/biomedicines14020311)
Supplement: Supplementary file 1 [file biomedicines-14-00311-s001.zip › Supplementary Figures.zip!/Fig S3.pdf]

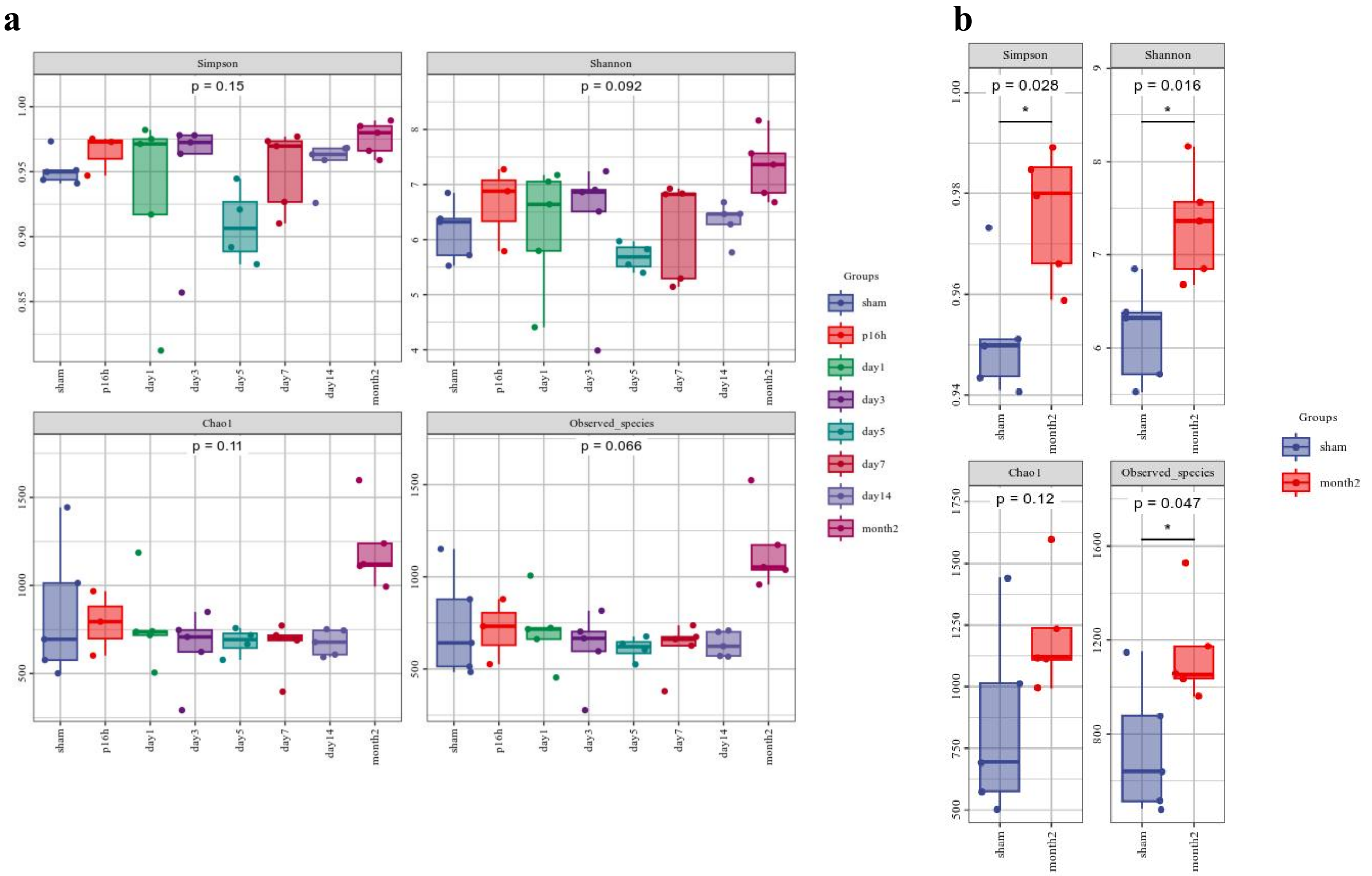

a
Simpson
p = 0.15
Shannon
p = 0.092
Chao1
p = 0.11
Observed_species
p = 0.066
sham
p16h
day1
day3
day5
day7
day14
month2
Groups
b
Simpson
p = 0.028
Shannon
p = 0.016
Chao1
p = 0.12
Observed_species
p = 0.047
Groups
sham
month2
