# Supplementary material for: Multi-Omics Profiling of mTBI-Induced Gut–Brain Axis Disruption: A Preliminary Study for Biomarker Screening and Mechanistic Exploration
Source: Biomedicines. 2026 Jan 30;14(2):311. doi: 10.3390/biomedicines14020311 (PMC12938811; doi:10.3390/biomedicines14020311)
Supplement: Supplementary file 1 [file biomedicines-14-00311-s001.zip › Supplementary Figures.zip!/Fig S4.pdf]

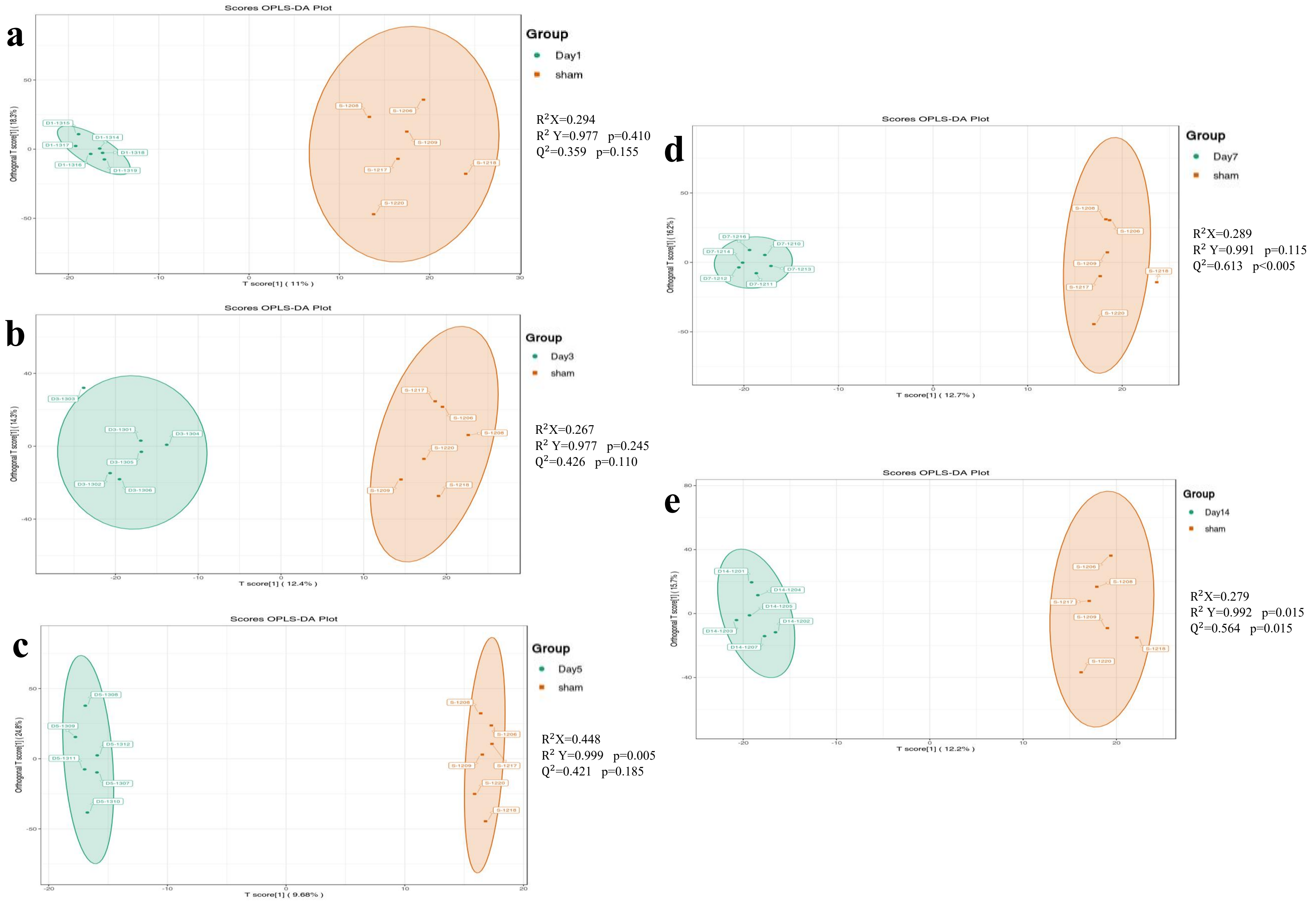

a
Scores OPLS-DA Plot
Group
Day1
sham
$R^2X$=0.294
$R^2$ Y=0.977 p=0.410
$Q^2$=0.359 p=0.155
Orthogonal T score[1] ( 18.3% )
T score[1] ( 11% )
b
Scores OPLS-DA Plot
Group
Day3
sham
$R^2X$=0.267
$R^2$ Y=0.977 p=0.245
$Q^2$=0.426 p=0.110
Orthogonal T score[1] ( 14.3% )
T score[1] ( 12.4% )
c
Scores OPLS-DA Plot
Group
Day5
sham
$R^2X$=0.448
$R^2$ Y=0.999 p=0.005
$Q^2$=0.421 p=0.185
Orthogonal T score[1] ( 24.8% )
T score[1] ( 9.68% )
d
Scores OPLS-DA Plot
Group
Day7
sham
$R^2X$=0.289
$R^2$ Y=0.991 p=0.115
$Q^2$=0.613 p<0.005
Orthogonal T score[1] ( 16.2% )
T score[1] ( 12.7% )
e
Scores OPLS-DA Plot
Group
Day14
sham
$R^2X$=0.279
$R^2$ Y=0.992 p=0.015
$Q^2$=0.564 p=0.015
Orthogonal T score[1] ( 15.7% )
T score[1] ( 12.2% )
