# Supplementary material for: Multi-Omics Profiling of mTBI-Induced Gut–Brain Axis Disruption: A Preliminary Study for Biomarker Screening and Mechanistic Exploration
Source: Biomedicines. 2026 Jan 30;14(2):311. doi: 10.3390/biomedicines14020311 (PMC12938811; doi:10.3390/biomedicines14020311)
Supplement: Supplementary file 1 [file biomedicines-14-00311-s001.zip › Supplementary Figures.zip!/Fig S5.pdf]

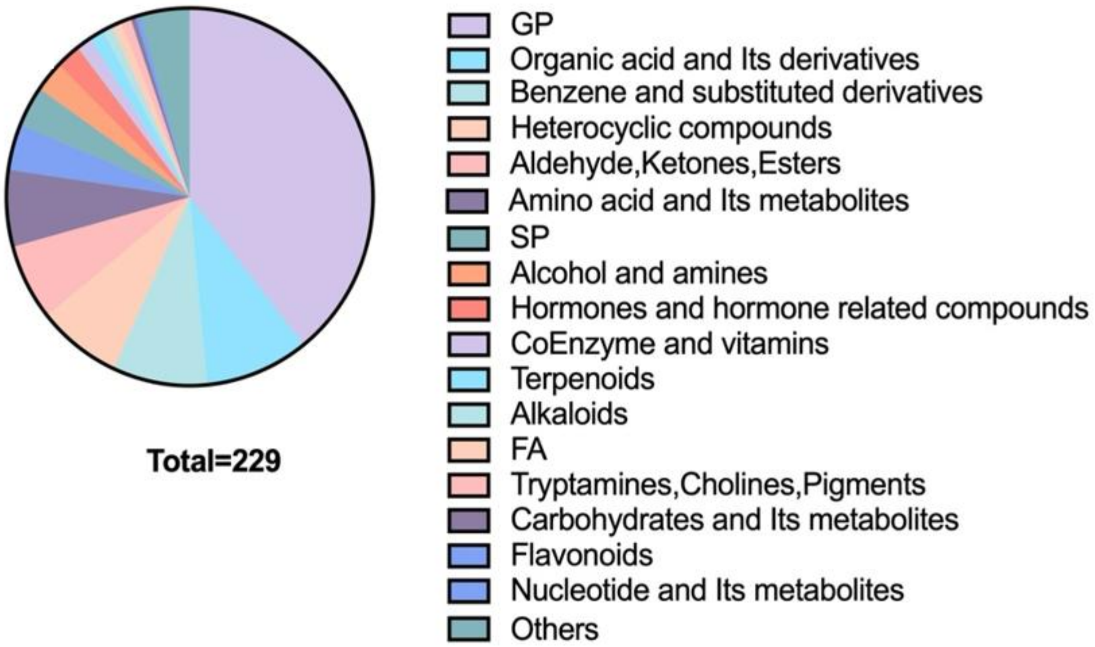
Total=229
GP
Organic acid and Its derivatives
Benzene and substituted derivatives
Heterocyclic compounds
Aldehyde,Ketones,Esters
Amino acid and Its metabolites
SP
Alcohol and amines
Hormones and hormone related compounds
CoEnzyme and vitamins
Terpenoids
Alkaloids
FA
Tryptamines,Cholines,Pigments
Carbohydrates and Its metabolites
Flavonoids
Nucleotide and Its metabolites
Others
