# Supplementary material for: Multi-Omics Profiling of mTBI-Induced Gut–Brain Axis Disruption: A Preliminary Study for Biomarker Screening and Mechanistic Exploration
Source: Biomedicines. 2026 Jan 30;14(2):311. doi: 10.3390/biomedicines14020311 (PMC12938811; doi:10.3390/biomedicines14020311)
Supplement: Supplementary file 1 [file biomedicines-14-00311-s001.zip › Supplementary Figures.zip!/Fig S6.pdf]

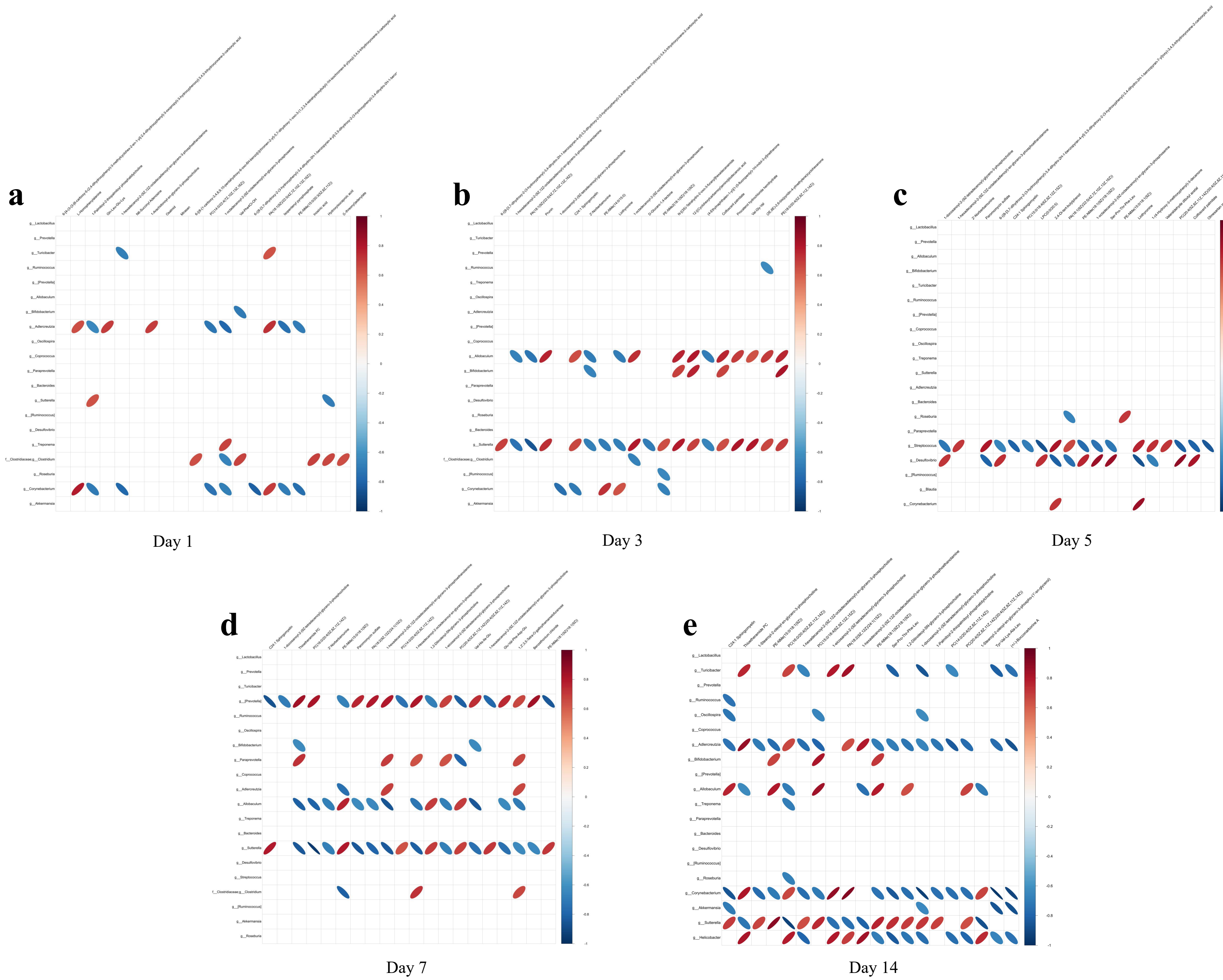

a
Day 1
b
Day 3
c
Day 5
d
Day 7
e
Day 14
