# Supplementary material for: Multi-Omics Profiling of mTBI-Induced Gut–Brain Axis Disruption: A Preliminary Study for Biomarker Screening and Mechanistic Exploration
Source: Biomedicines. 2026 Jan 30;14(2):311. doi: 10.3390/biomedicines14020311 (PMC12938811; doi:10.3390/biomedicines14020311)
Supplement: Supplementary file 1 [file biomedicines-14-00311-s001.zip › Supplementary Tables.zip!/Supplementary Tables.pdf]

Table S1. Condition of Liquid Chromatography

Time	A (%)	B (%)
0	95	5
2	80	20
5	40	60
6	1	99
7.5	1	99
7.6	95	5
10	95	5

Table S2 Conditions of AB TripleTOF6600 Mass Spectrometry

Name	ESI^+	ESI^-
Acquisition Time	10	10
Ionization Voltage（V）	5000	-4000
Ion Source Temperature（°C）	550	450
Nebulizer Gas(psi）	50	50
Auxiliary Gas（psi）	60	60
Curtain Gas（psi）	35	35
Declustering Voltage（V）	60	-60
MS1 Collision Energy（V）	10	-10
MS2 Collision Energy（V）	30	-30
Collision Energy Ramp（V）	15	15
MS1 Mass Range（Da）	50~1000	50~1000
MS2 Mass Range（Da）	25~1000	25~1000
MS1 Acquisition Time（s）	0.2s	0.2s
MS2 Acquisition Time（s）	0.04s	0.04s
Number of Candidate Ions	18	18

Table S3. Primer Sequences in RT-qPCR

Primers	Sequences（5'to 3'）
1-IL-1β-F	ACCGTGGCACATTCTGGTC
1-IL-1β-R	ATCCCATACACACGGACAACT
2-IL-10-F2	ACCCGGCATCTACTGGACT
2-IL-10-R2	TGGAGAGAGGTACAAACGAGG
3-TNF-α-F3	ATGTCGATGCCTGAGTGGAT
3-TNF-α-R3	TGTGCCTCAGGGAACAGTCT
4-Nsmaf-F	GAGCCATTTGTTGCCATCCAT
4-Nsmaf-R	GCCAGTGTTGTCCAATGTTCC
5-Cxcl1-F	GGGTGAGGAAATGTGTGAGAGG
5-Cxcl1-R	AGACGAGAAGGAGCATTGGT
β-actin-F	AGATCAAGATCATTGCTCCTCCT
β-actin-R	ACGCAGCTCAGTAACAGTCC

Table S4. Sequencing Yield of Gut Microbiota Samples in Weight-Drop Model

Sample name	group	Effective Tags	High-quality Sequences	ASV
FD0_1	sham	87509	53633	53556
FD0_2	sham	123925	66807	66414
FD0_4	sham	60498	38681	38620
FD0_5	sham	90094	52861	52675
FD0_6	sham	88484	57244	57163
F16h_4	p16	93037	52721	52559
F16h_5	p16	87334	61280	61212
F16h_6	p16	90742	54529	54399
F331_706	1d	68405	37899	37739
F331_707	1d	86642	48835	48756
F331_710	1d	59459	28818	28645
F331_712	1d	105581	51182	50916
F331_713	1d	59878	32993	32893
F402_706	3d	66359	39818	39748
F402_707	3d	65086	39443	39399
F402_710	3d	67407	36693	36591
F402_712	3d	62797	31787	31603
F402_713	3d	54502	33739	33685
FD5_2	5d	84517	56324	56207
FD5_4	5d	73675	53774	53719

FD5_5	5d	86856	53659	53538
FD5_6	5d	88251	55839	55749
F406_706	7d	60996	36888	36857
F406_707	7d	55059	29314	29185
F406_710	7d	88197	45063	44903
F406_712	7d	56004	31025	30938
F406_713	7d	57463	31582	31463
F413_706	14d	64814	36161	36093
F413_707	14d	73281	38920	38779
F413_710	14d	67639	37862	37716
F413_712	14d	67291	41372	41302
F413_713	14d	69764	40946	40832
F609_706	Month2	84822	48603	48481
F609_707	Month2	82830	40683	40486
F609_710	Month2	62065	37035	36951
F609_712	Month2	85452	38936	38667
F609_713	Month2	75700	40424	40224

* Sham, p16, 1d, 3d, 5d, 7d, 14d and Month2 correspond to fecal samples from controls, 16h post-injury, 1-day post-injury, 3-days post-injury, 5-days post-injury, 7-days post-injury, 14-days post-injury and 2-months post-injury, respectively.
